# Short-Term Effect of Temperature on Daily Emergency Visits for Acute Myocardial Infarction with Threshold Temperatures

**DOI:** 10.1371/journal.pone.0094070

**Published:** 2014-04-25

**Authors:** Suji Lee, Eunil Lee, Man Sik Park, Bo Yeon Kwon, Hana Kim, Dea Ho Jung, Kyung Hee Jo, Myung Ho Jeong, Seung-Woon Rha

**Affiliations:** 1 Department of Public Health, Graduate School, Korea University, Seoul, South Korea; 2 Department of Preventive Medicine, College of medicine, Korea University, Seoul, Korea; 3 Department of Statistics, Sungshin Women's University College of Natural sciences, Seoul, Korea; 4 Graduate School of Public Health, Graduate School, Korea University, Seoul, Korea; 5 Department of Cardiology, Chonnam National University Hospital, Gwangju, Korea; 6 Cardiovascular Center, Korea University Guro Hospital, Seoul, Korea; The Ohio State University, United States of America

## Abstract

**Background:**

The relationship between temperature and myocardial infarction has not been fully explained. In this study, we identified the threshold temperature and examined the relationship between temperature and emergency admissions due to MI in Korea.

**Methods:**

Poisson generalized additive model analyses were used to assess the short-term effects of temperature (mean, maximum, minimum, diurnal) on MI emergency visits, after controlling for meteorological variable and air pollution (PM10, NO_2_). We defined the threshold temperature when the inflection point showed a statistically significant difference in the regression coefficients of the generalized additive models (GAMs) analysis. The analysis was performed on the following subgroups: geographical region, gender, age (<75 years or ≥75 years), and MI status (STEMI or non-STEMI).

**Results:**

The threshold temperatures during heat exposure were for the maximum temperature as 25.5–31.5°C and for the mean temperature as 27.5–28.5°C. The threshold temperatures during cold exposure were for the minimum temperature as −2.5–1.5°C. Relative risks (RRs) of emergency visits above hot temperature thresholds ranged from 1.02 to 1.30 and those below cold temperature thresholds ranged from 1.01 to 1.05. We also observed increased RRs ranged from 1.02 to 1.65 of emergency visits when temperatures changes on a single day or on successive days.

**Conclusions:**

We found a relationship between temperature and MI occurrence during both heat and cold exposure at the threshold temperature. Diurnal temperature or temperature change on successive days also increased MI risk.

## Introduction

Myocardial infarction (MI) is a major social and health issue, because acute MI remains a leading cause of morbidity and mortality worldwide [1]. A number of studies showed that cold temperature is associated with the increased occurrence of MI due to an increase in plasma viscosity and serum cholesterol levels, blood pressure, sympathetic nervous activities, and platelet aggregation [2]–[4]. Heat exposure is also reported to be associated with such physiological changes as increases in heart rate, blood viscosity, and coagulability [5], which could be risk factors for MI. However, only a few studies supporting heat exposure to MI have been published [6], [7].

According to the Intergovernmental Panel on Climate Change, climate conditions have become more variable with more extreme heat episodes, unpredictable weather, including sudden cold, hot, wet, or dry spells, and extreme weather events, including floods and droughts [8]. With climate change and a rapidly growing elderly population throughout the world, MI mortality from extreme heat and cold weather events is a significant public burden that may worsen in the future. In additions, an individual's susceptibility may be exacerbated by underlying chronic medical conditions and drug treatments that affect the body's capacity to adapt to temperature changes [9]. Therefore, temperature-associated episodes of MI may increase with aggravated climate conditions, especially in older people, those with underlying cardiovascular diseases, and those who are poor, uneducated, or isolated [10]–[12].

From a public health perspective, the identification of population subgroups vulnerable to heat and cold is important for effective prevention, and clinicians also should be aware that exposure to environmental heat and cold is a risk factor for MI and should consider this for risk prevention and management [13].

Public concerns about temperature-associated diseases began from thousands of heat-related deaths in Europe in 2003; one of the most important ways to prevent heat stroke is to establish a public warning system based on a threshold temperature above which heat stroke may increase rapidly [14]. A number of studies have examined the influence of meteorological factors and seasonal variations on MI morbidity and mortality with lag effect, However, no warning systems for temperature-related MI have been reported. Dilaveris et al. show that a minimum rate of MI occurred at a temperature of 23.3°C, with the rate of MI increasing both above and below this temperature [15]. Rossi et al. reported that high temperature(above 27°C) is also associated MI mortality on the same day [16]. The Myocardial Ischaemia National Audit Project (MINAP) registry study showed linearity only between cold temperature and MI without threshold temperatures [17]. And the threshold temperatures for diurnal temperature change(DTR) or successive daily temperature changes (SDTC) are also needed because these temperature variations are reported as the important risk factors for MI [14], [18], [19].

Therefore, we evaluated the effects of hot, cold, and DTR and SDTC on the number of emergency visits for MI with threshold temperatures according to geographical area, age, sex, and severity of MI by using the Korea Working Group of Myocardial Infarction (KorMI) data. KorMI was established in November 2005 as a Korean prospective multi-center on-line registry for investigating the risk factors of mortality in acute MI patients, and registry data are based on nationwide hospital emergency visits with the support of the Korean Circulation Society [20]. We also estimated risk ratios by a 1°C change above or below the thresholds using generalized additive models (GAMs). We demonstrated that threshold temperatures were different according to geographical locations with modifying seasonal effect. In addition, we also found that DTR in the spring, autumn, and winter, and SDTC in the spring increased MI risk.

## Methods

### Ethics Statement

This study was performed with the support of the Korean Circulation Society (KCS) to commemorate the 50^th^ Anniversary of the KCS. The authors of this manuscript have certified that the information contained herein is true and correct as reflected in the records of the KUGH Medical Device Institutional Review Board.

### Study Area

South Korea is located in the southern part of the Korean peninsula, including all its islands, lying between latitudes 33° and 39°N, and longitudes 124° and 130°E. Its total area is 100,032 square kilometers (38,622.57 square miles) [21]. Approximately 50 million residents lived in Korea in 2011. South Korea tends to have both a humid continental and subtropical climate, and is affected by the East Asian monsoon [22]. South Korea has four distinct seasons: spring, summer, autumn, and winter. Winter temperatures were higher along the southern coast and considerably lower in the mountainous interior. Summer can be uncomfortably hot and humid, with temperatures exceeding 30°C (86°F) in most parts of the country with heavy rainfall [23]. The weather of South Korea differs between the central and southern parts of the Korean peninsula, where the southern part is warmer than the central part.

### Data

The KorMI registry covers a total of 62 general hospitals located in 16 major cities in Korea. We excluded data from the hospital on the Jeju island, which is located about 100 kilometers (60 miles) off the southern coast of the Korean peninsula, because the weather conditions on the island are quite different from those on the peninsula. We also excluded data from two hospitals in Gangwon Province, because the number of patients was not large enough to represent the area. We analyzed patient data from January 1, 2006 to December 31, 2010. The average number of MI patients who visited the emergency room was 4,564 per year. We defined the first medical contract time as the occurrence time of MI and used KorMI data for the statistical analysis, such as history of hypertension and diabetes, age, gender, and MI status, including ST-segment elevation myocardial infarction (STEMI) and non-STEMI.

We obtained weather data from the Korean Meteorological Administration. Data included daily mean, minimum, and maximum temperatures; DTR; SDTC; daily precipitation; humidity; dew point; sea level pressure; and wind speed for the study time period. Air pollution data, including ambient 24-h average concentrations of PM10, NO_2_, SO_2_, ozone, and CO, were provided by the National Institute of Environmental Research, Korea.

### Statistical Analysis

#### MI-temperature Plotting

To calculate the average daily adjusted emergency visits (DAEVs) for MI according to daily temperature or temperature change, we divided the total number of daily emergency visits that occurred on all days with a specific temperature (value was calculated for each 1°C temperature along the range from -X°C to X°C), the numerator, by the total number of days that temperature occurred over the X-day study period, the denominator. The DAEVs were plotted for each 1°C range, and piecewise regression (PR) analysis was applied to find the inflection points of the relationship between DAEVs and temperature with maximum R^2^ values. The best fit was judged on the basis of the residual sum of squares and the value of the R^2^ statistic by PR analysis.

#### Poisson GAM with Threshold Effect

We estimated the temperature- daily emergency visits relationship using generalized additive models (GAMs) with nonparametric smoothing functions (splines) to describe nonlinear relations [24]. Temperature variables were the mean, minimum, maximum temperature, DTR, SDTC. Moreover, to observe the independent effects of temperature on emergency visits for MI, we controlled for potential confounders, such as humidity, sea level pressure, and air pollutants (PM10, NO_2_). To estimate the lag effect of the temperature- daily emergency visits relationship, temperature variables of the previous seven days were applied to the GAM using single-day lags from lag 0 (current day) to lag 7 (7 days before the event day).

We defined the threshold temperature when the inflection point showed a statistically significant difference in the regression coefficients of the GAM analysis between the temperature ranges above the inflection point and below the point. Each temperature range was treated as dummy variables. The relationship between the daily emergency visits for MI and temperature was also analyzed by subgroups in the following categories: geographical region, gender, age (under and over 75 years), and MI status (either STEMI or non-STEMI). Seasonal effects were analyzed especially for the relationships between daily emergency visits and DTR, or DAEV and SDTC.

## Results

The total number of emergency visits for MI was 27,388 during the 5-year study period, and the general characteristics of subjects in the central region differed from those in the southern region (Table 1). The number of male MI patients was greater than female patients in both regions; however, the proportion of female patients was larger in the southern region (30.7%) than in the central region (27.8%). And the weather in the southern region was warmer than that of the central region (Table 2, File S1).

**Table 1:** General Characteristics of the Study Subjects.

	Central region	Southern region	All	P-value
Total MI	12,586	14,802	27,388	
Age, mean±SD	63.1±13.2	63.9±12.7	63.5±12.6	
Male, % (n)	72.1.7 (8,377)	69.3 (9,069)	70.5 (20,694)	<0.001
Female, % (n)	27.8 (3,239)	30.7 (4,022)	29.5 (8,643)	
STEMI, % (n)	60.1 (6,914)	55.0 (7,094)	57.0 (16,285)	<0.001
Non-STEMI, % (n)	40.2 (6,914)	45.0 (5,802)	42.2 (12,242)	
History of Hypertension % (n)	51.5 (5,934)	47.7 (6,218)	50.4 (14,407)	<0.001
History of Diabetes mellitus % (n)	27.6 (3,180)	27.2 (3,547)	27.8 (7,958)	0.2217

MI: Myocardial infarction.

STEMI: ST elevation myocardial infarction.

Non-STEMI: Non-ST elevation myocardial infarction.

**Table 2:** Summary Statistics for Temperature and other Meteorological Variables with the Level of Air pollutants in Study Areas.

	Central region				Southern region				Combined regions
Parameter	Mean (SD)	Range	Median	IQR	Mean (SD)	Range	Median	IQR	Mean (SD)
**Temperature**									
Mean (°C)	12.72 (10.18)	−13.2–30.7	14.1	17.8	14.32 (9.13)	−8.0–31.5	15.3	15.5	13.32 (9.83)
Minimum (°C)	8.74 (10.43)	−19.5–27.1	9.4	18.3	10.22 (9.57)	−15.3–28.2	10.6	16.7	9.3 (10.14)
Maximum (°C)	17.31 (10.32)	−10.7–36.2	19.2	17.9	19.3 (9.14)	−4.3–37.7	20.6	15.1	18.06 (9.94)
Diurnal temperature (°C)	8.57 (3.16)	1.0–24.0	8.4	4.1	9.09 (3.63)	0.8–25.7	8.7	4.8	8.77 (3.35)
**Other meteorological variables**									
Precipitation (mm)	4.03 (15.30)	0.0–272.5	0.0	0.5	3.5 (13.25)	0.0–310	0.0	0.5	3.83 (14.55)
Precipitation (log mm)	−0.004 (1.58)	−3.0–5.61	0.0	0.0	0.14 (1.39)	−3.0–5.74	0.0	0.0	0.05 (1.51)
Relative humidity (%)	63.72 (14.76)	19.9–99.0	64.6	21.7	63.68 (16.73)	16.1–99.0	65.5	24.4	63.7 (15.53)
Sea-level pressure (hPa)	1015.98 (8.04)	993.8–1038.2	1016.1	12.5	1015.89 (7.51)	993.8–1038.4	1016	11.4	1015.95 (7.84)
**Level of air pollutants**									
PM10 (µg/m^3^)	55.26 (37.76)	0.0–1153.25	48.09	36.21	52.29 (34.15)	0.0–974.0	45.95	33.0	54.14 (36.47)
NO_2_ (ppm)	0.03 (0.01)	0.0–0.14	0.03	0.02	0.02 (0.01)	0.0–0.12	0.02	0.01	0.03 (0.01)
CO (ppm)	0.63 (0.32)	0.0–4.7	0.56	0.35	0.52 (0.30)	0.0–3.37	0.46	0.36	0.59 (0.31)
SO_2_ (ppb)	5.59 (3.09)	0.0–33.87	4.96	3.74	5.81 (3.91)	0.0–45.78	4.71	4.08	5.67 (3.42)
O_3_ (ppm)	0.02 (0.07)	0.0–0.05	0.02	0.02	0.02 (0.01)	0.0–0.09	0.02	0.02	0.02 (0.06)

SD: Standard deviation.

IQR: Interquartile range.

**Figure 1:**
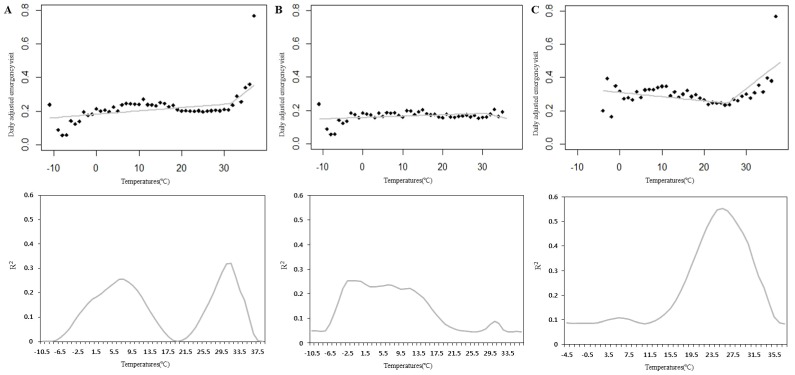
Daily adjusted emergency visit (DAEV) rate for MI according to maximum temperature by regions: A. Combined regions, B. Central region, C. Southern region. Lower figures showed change of R^2^ values in each temperature by piecewise analysis and maximum R^2^ value was chosen as the inflection point. The maximum R^2^ value of the central region was 30.5°C; however, it did not show a threshold effect.

DAEVs for MI were plotted according to the daily maximum temperature for the central and southern regions combined (Figure 1A–1C). PR analysis revealed that the peak values indicated a significant change point. In the plot of combined regions, 31.5°C was identified for the inflection point for sudden increases in MI emergency visits. The southern region showed a prominent inflection point at 25.5°C with threshold effect. Meanwhile, the inflection point of the central region was 30.5°C, however, it did not show a threshold effect (Table 3). DAEVs according to the daily mean and minimum temperatures were plotted to find threshold temperatures (data not shown).

**Table 3:** Relative Risk of Myocardial Infarction per 1°C Change in Temperature above Threshold temperature by Subgroup.

Temperature(°C)	Threshold (°C)^f^	Lag (days)	RR (95% CI)
***Effect of heat*** a			
**Maximum** c			
All	31.5*	4	1.07 (1.05–1.10)
Central	-g	-	-
Southern	25.5**	4	1.02 (1.00–1.03)
≥75 years	31.5*	4	1.12 (1.06–1.18)
≤75 years	31.5*	4	1.06 (1.03–1.09)
Male	30.5**	0	1.08 (1.04–1.12)
Female	-	-	-
STEMI	30.5**	4	1.06 (1.01–1.11)
Non-STEMI	31.5*	0	1.10 (1.02–1.19)
**Mean** d			
All	28.5*	0	1.26 (1.08–1.46)
Central	-	-	-
Southern	27.5*	0	1.12 (1.01–1.24)
≥75years	-	-	-
≤75years	28.5*	0	1.24 (1.04–1.47)
Male	28.5*	0	1.28 (1.07–1.53)
Female	-	-	-
STEMI	28.5*	0	1.23 (1.00–1.50)
Non-STEMI	28.5*	0	1.30 (1.03–1.64)
***Effect of cold*** b			
**Minimum** e			
All	−1.5**	5	1.01 (1.00–1.02)
Central	−1.5**	1	1.04 (0.99–1.11)
Southern	−2.5*	5	1.05 (1.02–1.08)
≥75years	−1.5**	1	1.03 (1.00–1.06)
≤75 years	1.5**	5	1.01 (1.00–1.02)
Male	−1.5**	5	1.01 (1.00–1.03)
Female	−1.5**	5	1.02 (1.00–1.05)
STEMI	−1.5**	1	1.01 (0.99–1.02)
Non- STEMI	−1.5**	5	1.02 (1.00–1.04)

Model adjusted for precipitation, humidity, sea level pressure, and air pollutants (PM10, NO_2_) using a spline function.

RR = Relative risk.

STEMI: ST elevation myocardial infarction.

Non-STEMI: Non-ST elevation myocardial infarction.

Spring: March–May, Summer: June–August, Autumn: September–November, Winter: December–February.

*P<0.05;

** ***P***<0.001.

a For heat exposure, temperature increase of 1°C above threshold.

b For cold exposure, temperature decrease of 1°C below threshold.

c Maximum temperature.

d Mean temperature.

e Minimum temperature.

f Threshold temperature.

g No threshold effect was identified.

The threshold temperatures during heat exposure were for the maximum temperature as 25.5–31.5°C and for the mean temperature as 27.5–28.5°C. The threshold temperatures during cold exposure were −2.5–1.5°C.

A significant increased risk for MI with heat exposure was found above the threshold temperature both at the maximum and mean temperatures (Table 3). The RR of MI per a 1°C change in the maximum temperature was lowest in the southern region (RR = 1.02), and the RR in the old age group was greatest (RR = 1.12). Most subgroups showed a 4lag-day effects; however, male and non-STEMI subjects showed immediate daily temperature effects on MI with 0 lag-day effects. The RRs from mean temperatures were relatively higher than those from maximum temperatures. The RR of MI per a 1°C change in the mean temperature was lowest in the southern region (RR = 1.12), and the RR in the non-STEMI group was highest (RR = 1.30). Lag effects were shown on the current day. Increased risk of MI visits was also found below the threshold temperature from the minimum temperature. The RRs associated with the minimum temperature ranged from 1.01 to 1.05. In contrast to exposure at higher temperatures, the RR of MI per a 1°C change in the minimum threshold temperature was greatest in the southern region (RR = 1.05).

Both the range of temperatures on a single day or on successive days showed increased MI risk (Tables 4 and 5). The DTR above 7.5 or 8.5°C in the spring and autumn showed threshold effects for increased MI visits. Non-STEMI patients in the spring showed an increased risk for MI above 6.5°C of the DTR. The threshold temperature of DTR in the winter was 4.5 to 6.5°C, which was lower than in the spring or autumn. Lag-day effects for DTR were 1 or 2 days; however, delayed lag effects were evident in males at 7 days and in the old age group at 4 days in winter (Table 4).

**Table 4:** Relative Risk of Myocardial Infarction per 1°C Change in Diurnal Temperature Range (DTR) above the Threshold Temperature in All Regions by Season.

	Spring			Autumn			Winter		
DTR (°C)	Threshold(°C)^a^	Lag (days)	RR (95% CI)	Threshold (°C)^a^	Lag (days)	RR (95% CI)	Threshold (°C)^a^	Lag (days)	RR (95% CI)
**All**	7.5**	1	1.03 (1.02–1.04)	-	-	-	6.5**	1	1.02 (1.01–1.03)
**Central region**	8.5**	1	1.03 (1.01–1.05)	7.5**	2	1.02 (1.01–1.04)	6.5**	1	1.02 (1.00–1.04)
**Southern region**	-b	-	-	-	-	-	-	-	-
**Male**	7.5**	1	1.03 (1.02–1.04)	-			6.5**	7	1.02 (1.01–1.03)
**Female**	7.5**	1	1.04 (1.03–1.06)	8.5**	2	1.04 (1.01–1.06)	6.5**	1	1.03 (1.01–1.05)
**≥75 years**	7.5**	1	1.03 (1.01–1.05)	7.5**	2	1.03 (1.01–1.06)	6.5**	4	1.02 (1.00–1.05)
**≤75 years**	7.5**	1	1.03 (1.02–1.05)	-	-	-	5.5**	1	1.02 (1.01–1.04)
**STEMI**	8.5**	1	1.03 (1.01–1.04)	7.5**	2	1.02 (1.01–1.04)	7.5**	1	1.03 (1.01–1.04)
**Non-STEMI**	6.5**	1	1.03 (1.02–1.05)	-	-	-	4.5**	1	1.02 (1.00–1.03)

Model adjusted for precipitation, humidity, sea level pressure, and air pollutants (PM10, NO_2_) using a spline function.

RR = Relative risk.

STEMI: ST elevation myocardial infarction.

Non-STEMI: Non-ST elevation myocardial infarction.

Spring: March–May, Summer: June–August, Autumn: September–November, Winter: December–February.

*P<0.05;

** ***P***<0.001.

a Threshold temperature.

b No threshold effect was identified.

**Table 5:** Relative Risk of Myocardial Infarction per 1°C Change in Successive Daily Temperature Changes by Subgroup.

	Spring			
Successive daily temperature changes (°C)	Threshold (°C)^a^	RR (95% CI)	Threshold (°C)^b^	RR (95% CI)
All	4.5**	1.20 (1.01–1.43)*	−4.5**	1.10 (1.02–1.18)*
Central region	4.5**	1.65 (1.01–2.70)*	−7.0**	1.30 (1.04–1.62)*
South region	-c	-	−6.5**	1.49 (1.23–1.81)*
Male	4.5**	1.26 (1.03–1.55)*	−4.5**	1.09 (1.00–1.20)
Female	-	-	−4.5**	1.12 (0.98–1.28)
≥75 years	-	-	-	-
≤75 years	4.5**	1.31 (1.08–1.60)*	−4.5**	1.09 (1.00–1.18)*
STEMI	4.5**	1.30 (1.05–1.60)*	−4.5**	1.15 (1.04–1.26)*
Non-STEMI	-	-	-	-

Model adjusted for precipitation, humidity, sea level pressure, and air pollutants (PM10, NO_2_) using a spline function.

RR = Relative risk.

STEMI: ST elevation myocardial infarction.

Non-STEMI: Non-ST elevation myocardial infarction.

Spring: March–May, Summer: June–August, Autumn: September–November, Winter: December–February.

*P<0.05;

** ***P***<0.001.

a Temperature rise between consecutive days.

b Temperature fall between consecutive days.

c No threshold effect was identified.

Increases and decreases in successive daily mean temperatures showed significant effects only in the spring (Table 5). Significant increases in MI risk with a 4.5°C increase in temperature was evident in several subgroups, including the central region, males, the young age group, and STEMI patients. A decrease in temperature over 4.5°C showed significant effects among all subgroups except for old age and non-STEMI patients.

## Discussion

Many reports showed various threshold temperatures using different estimation methods; however, they did not estimate the actual threshold temperatures that would show an increase in the risk of MI. Most reports used a Poisson regression model with a natural spline function to estimate the relationship between temperature and risk of MI [18], [25], [26]. But the spline function in GAM analysis cannot provide an exact inflection point. Therefore, PR analysis after plotting temperature and DEAV would be a better approach for identifying the threshold temperature. We calculated the threshold temperature using regression coefficients from dummy variables (both below and above the inflection point) of the GAM analysis after finding the inflection point from the PR analysis. We estimated threshold temperatures in all subgroups. Several subgroups showed no threshold temperature, but most subgroups showed similar threshold temperatures for maximum temperature (25.5 to 31.5°C) and mean temperature (27.5 to 28.5°C). In the southern region, the threshold temperature was relatively lower than that for most other groups (25.5°C), and the rate of MI increased rapidly over 32°C(Figure 1C). These findings suggest that patients in the southern region are vulnerable to higher temperatures and temperature changes above the threshold temperature.

Many reports showed various threshold temperatures each using different estimation methods, or showed no threshold temperature. The Myocardial Ischemia National Audit Project registry study in England and Wales showed linearity between cold temperature and MI, but not with threshold temperature [17]. Dilaveris et al. [15] showed that the rate of MI events increased smoothly both above and below the minimum event rate at 23.3°C based on the U-shape of the MI mortality and temperature association, which showed a similar pattern to our results in the southern region. Rossi et al. [16] reported an increase in MI mortality above 27°C compared to MI mortality when 14°C was the reference temperature. Gasparrini et al. [27] used the 93^rd^ percentile of year-round maximum temperature as the threshold temperature, including 20.9 to 24.7°C, based on the region.

The highest RRs by mean temperature were found for males, the young age group, and non-STEMI patients who may participate in many outdoor activities when compared with other subgroups. These findings suggest that outdoor daily activity is strongly related to the effects of temperature on MI risk. Consistent with our findings, Na et al. [28] found that heat-related illnesses largely influence the age group from 20 to 64 years in Korea. Goggins et al. and Morabito et al. [29], [30] also found a higher RR in males than in females in Italia City and Taiwan, respectively. However, Bhaskaran et al. [31] reported that only STEMI patients showed a significant RR above 20°C. This inconsistency between our results and Bhaskaran's report may be explained by the difference in average temperatures in the summer and the outdoor activity of the study population.

Many studies reported a cold effect on MI when temperatures were above the freezing temperature. Wang et al. [32] reported that daily MI events occurred more frequently below 10°C compared with above 20°C in Hiroshima, Japan, and MI admission increased in Hong Kong per 1°C drop below a mean temperature of 24°C [29]. MI mortality increased in the USA under a maximum temperature of 17°C [33]. However, several studies showed that a threshold temperature of −1.5°C or −2.5°C for MI risk were similar to our study just below 0°C [34], [35]. The differences in cold threshold temperature were related with the diverse weather in each country.

Additionally, the RR of the cold effect ranged from 1% (95% CI: 0.2%–2.4%) to 5% (95% CI: 2.0%–7.9%) below the threshold temperature in our study, which is larger than that seen in other studies [17]. A multi-country study using World Health Organization data found increases in age-standardized MI rates between 0.1% and 2.3% per a 1°C drop across 24 locations [36]. This report showed general cold effects without using threshold temperature.

We found increased risks of MI with DTR in spring, autumn, and winter, but not in summer. DTR changes over 6.5–8.5°C increased MI risk in the spring and those over 4.5–7.5°C increased MI risk in the winter, suggesting an increased risk of MI due to relatively small changes in DTR in the winter, especially for non-STEMI patients. These findings may explain the higher risk of MI in the winter, similar to the results of other studies [37], [38]. Ebi et al. [14] reported that changes in the daily maximum and minimum temperatures resulted in increased hospitalizations of elderly people for MI by 6–13% in several American cities.

We also found that the difference in mean temperature between successive days either rise or fall, related to increased MI risk. Messner et al. [18] reported similarly that increases in temperature between consecutive days are associated with increases in MI hospitalization. We also found an increased risk of MI only in the spring. This risk was especially apparent for STEMI patients and in the younger age group (<75 years). No other reports found an increased risk of MI in the spring because of SDTC.

Our study showed a U-shaped association between temperature and MI risk, including both hot and cold effects, which is consistent with several previously published studies [15], [25], [33]. In addition, we found that diurnal temperature or temperature change on successive days also increased MI risk. We could not adjust for other confounding factors, including indoor temperature, outdoor daily activity, smoking and other behavioral factors, socioeconomic status, air conditioning of the house or working site, and effects of other pre-existing diseases. Despite these limitations, our study provides useful information about actual threshold temperatures with regard to RR of MI and could be used to establish a warning system for MI in hot and cold temperatures.

In conclusion, climate change, including extreme weather or increases in average temperatures, may increase the risk of MI in susceptible populations. Our findings provide useful information for identifying the risk of MI in vulnerable groups for establishing climate change adaptation strategies.

## Supporting Information

File S1 Summary of Monthly Average Temperature and Number of Emergency visits for Myocardial Infarction in the Central and Southern regions: Table S1. Summary Statistics of Central region for Temperature and other Meteorological Variables with the Level of Air pollutants by Season: Table S2. Summary Statistics of Southern region for Temperature and other Meteorological Variables with the Level of Air pollutants by Season: Table S3.
(DOCX)
