# Supplementary material for: Short-Term Effect of Temperature on Daily Emergency Visits for Acute Myocardial Infarction with Threshold Temperatures
Source: PLoS One. 2014 Apr 25;9(4):e94070. doi: 10.1371/journal.pone.0094070 (PMC4000206; doi:10.1371/journal.pone.0094070)
Supplement: File S1 — Summary of Monthly Average Temperature and Number of Emergency visits for Myocardial Infarction in the Central and Southern regions: Table S1. Summary Statistics of Central region for Temperature and other Meteorological Variables with the Level of Air pollutants by Season: Table S2. Summary Statistics of Southern region for Temperature and other Meteorological Variables with the Level of Air pollutants by Season: Table S3. (DOCX) [file pone.0094070.s001.docx]

**Table S1. Summary of Monthly Average Temperature and Number of Emergency visits for Myocardial Infarction in the Central and Southern regions**

| **Month** | **Number of Emergency visits** | |  | **Central region** | | | | | |  | **Southern region** | | | | | |
| --- | --- | --- | --- | --- | --- | --- | --- | --- | --- | --- | --- | --- | --- | --- | --- | --- |
|  | **Central**  **region** | **Southern region** |  | **Mean**^a^ | **Max**^b^ | **Min**^c^ | **DTR** | **SDTC^d^** | **SDTC^e^** |  | **Mean**^a^ | **Max**^b^ | **Min**^c^ | **DTR** | **SDTC^d^** | **SDTC^e^** |
| January | 1123 | 1393 |  | -1.6 | 2.7 | -5.4 | 8.1 | 8.2 | -7.9 |  | 1.8 | 6.6 | -2.1 | 8.7 | 8.8 | -8.6 |
| February | 1052 | 1316 |  | 0.9 | 5.4 | -3.1 | 8.6 | 8.7 | -8.4 |  | 3.8 | 8.8 | -0.5 | 9.4 | 9.5 | -9.2 |
| March | 1126 | 1405 |  | 5.6 | 10.5 | 1.3 | 9.2 | 9.3 | -9.1 |  | 7.7 | 13.2 | 3.0 | 10.1 | 10.3 | -10.0 |
| April | 1139 | 1255 |  | 12.0 | 17.3 | 7.2 | 10.0 | 10.3 | -9.7 |  | 13.1 | 18.8 | 8.1 | 10.7 | 10.9 | -10.4 |
| May | 1087 | 1145 |  | 17.9 | 23.2 | 13.1 | 10.2 | 10.2 | -10.0 |  | 18.3 | 24.0 | 13.5 | 10.5 | 10.6 | -10.5 |
| June | 1023 | 1153 |  | 22.3 | 27.0 | 18.3 | 8.7 | 8.9 | -8.6 |  | 22.3 | 27.0 | 18.3 | 8.7 | 8.9 | -8.6 |
| July | 989 | 1138 |  | 24.6 | 28.1 | 21.7 | 6.4 | 6.5 | -6.3 |  | 25.0 | 28.9 | 22.2 | 6.7 | 6.8 | -6.6 |
| August | 971 | 1168 |  | 25.9 | 29.8 | 22.8 | 7.1 | 6.9 | -7.1 |  | 26.5 | 30.8 | 23.4 | 7.4 | 7.3 | -7.5 |
| September | 956 | 1053 |  | 21.6 | 26.0 | 17.8 | 8.2 | 8.0 | -8.4 |  | 22.4 | 26.9 | 18.9 | 8.0 | 7.8 | -8.1 |
| October | 916 | 1124 |  | 15.5 | 20.7 | 11.1 | 9.6 | 9.4 | -9.8 |  | 17.1 | 22.7 | 12.7 | 10.0 | 9.8 | -10.1 |
| November | 1115 | 1316 |  | 7.5 | 12.2 | 3.3 | 8.9 | 8.7 | -9.2 |  | 9.9 | 15.2 | 5.4 | 9.9 | 9.7 | -10.2 |
| December | 1089 | 1336 |  | -0.1 | 4.1 | -3.9 | 8.1 | 7.8 | -8.4 |  | 3.2 | 8.1 | -0.9 | 9.0 | 8.8 | -9.3 |

DTR: Diurnal temperature change

SDTC: Successive daily temperature change

**^a^** Mean temperature

**^b^** Maximum temperature

**^c^** Minimum temperature

^d^ Temperature rise between consecutive days

^e^ Temperature fall between consecutive day

**Table S2. Summary Statistics of Central region for Temperature and other Meteorological Variables with the Level of Air pollutants by Season**

| **Parameter** | **Spring** | | | |  | **Summer** | | | |  |  | **Fall** | | | |  |  | **Winter** | | | |
| --- | --- | --- | --- | --- | --- | --- | --- | --- | --- | --- | --- | --- | --- | --- | --- | --- | --- | --- | --- | --- | --- |
|  | **Mean (SD)** | **Range** | **Median** | **IQR** |  | **Mean (SD)** | **Range** | **Median** | **IQR** |  |  | **Mean (SD)** | **Range** | **Median** | **IQR** |  |  | **Mean (SD)** | **Range** | **Median** | **IQR** |
| **Temperature** |  |  |  |  |  |  |  |  |  |  |  |  |  |  |  |  |  |  |  |  |  |
| Mean (°C) | 11.82(6.0) | -5-24.3 | 12.1 | 9.6 |  | 24.26(2.61) | 14.9-30.7 | 24.5 | 3.5 |  |  | 14.86(6.73) | -4.2-28.7 | 15.8 | 10.9 |  |  | -0.3(4.44) | -13.2-14 | -0.1 | 6.5 |
| Minimum (°C) | 7.20(5.85) | -9.5-19.2 | 7.4 | 9.2 |  | 20.96(2.87) | 11.2-27.1 | 21.2 | 4.2 |  |  | 10.74(7.03) | -8-25.3 | 11.4 | 11.3 |  |  | -4.19(4.6) | -19.5-12.5 | -4.2 | 6.6 |
| Maximum (°C) | 16.99(6.66) | -1.6-32.7 | 17.2 | 10.3 |  | 28.34(3.09) | 17-36.2 | 28.6 | 4.2 |  |  | 19.65(6.87) | -0.7-34.3 | 20.7 | 10.3 |  |  | 4.03(4.8) | -10.7-20 | 4.3 | 6.6 |
| Diurnal temperature (°C) | 9.79(3.51) | 1.7-24 | 9.7 | 4.6 |  | 7.38(2.71) | 1.4-17.1 | 7.3 | 3.7 |  |  | 8.91(2.97) | 1.1-19.4 | 8.8 | 3.7 |  |  | 8.22(2.84) | 1-19.9 | 7.9 | 3.5 |
| **Other meteorological variables** |  |  |  |  |  |  |  |  |  |  |  |  |  |  |  |  |  |  |  |  |  |
| Precipitation (mm) | 2.51(7.87) | 0.0-93 | 0.0 | 0.5 |  | 9.51(24.92) | 0-272.5 | 0.05 | 6 |  |  | 3.24(13.93) | 0-259.5 | 0.0 | 0.2 |  |  | 0.74(2.86) | 0-46.5 | 0.0 | 0.05 |
| Precipitation (log mm) | -0.06(1.47) | -3.0-4.53 | 0.0 | 0.0 |  | 0.45(1.94) | -3.0-5.61 | 0.0 | 1.7 |  |  | -0.03(1.45) | -3.0-5.56 | 0.0 | 0.0 |  |  | -0.38(1.26) | -3.0-3.84 | 0.0 | 0.0 |
| Relative humidity (%) | 57.84(14.85) | 19.9-98.8 | 57 | 20.8 |  | 73.32(11.49) | 24-98.8 | 74 | 14.4 |  |  | 65.27(12.65) | 25.6-99 | 66 | 17.5 |  |  | 58.34(14.15) | 26.6-99 | 57.85 | 21 |
| Sea-level pressure (hPa) | 1014.76(6.33) | 993.8-1031 | 1014.8 | 8.6 |  | 1007.35(4.23) | 995.7-1018.8 | 1007.5 | 6.4 |  |  | 1018.13(5.48) | 1003-1032.8 | 1018.1 | 7.4 |  |  | 1023.85(5.3) | 998.6-1038.2 | 1024.2 | 7.1 |
| **Level of air pollutants** |  |  |  |  |  |  |  |  |  |  |  |  |  |  |  |  |  |  |  |  |  |
| PM10 (µg/m^3^) | 67.49(51.76) | 0.0-1153.25 | 57.74 | 37.41 |  | 43.89(25.76) | 3.0-203.21 | 39.07 | 32.32 |  |  | 48.81(30.47) | 3.87-324.92 | 41.33 | 32.64 |  |  | 60.91(32.71) | 0.0-369.42 | 53.21 | 35.38 |
| NO_2_ (ppm) | 0.03(0.01) | 0.0-0.1 | 0.03 | 0.02 |  | 0.03(0.01) | 0.0-0.09 | 0.02 | 0.02 |  |  | 0.03(0.01) | 0.0-0.14 | 0.03 | 0.02 |  |  | 0.04(0.02) | 0.0-0.11 | 0.04 | 0.02 |
| CO (ppm) | 0.58(0.23) | 0.0-2.14 | 0.55 | 0.29 |  | 0.47(0.17) | 0.0-1.84 | 0.45 | 0.22 |  |  | 0.63(0.29) | 0.05-2.86 | 0.58 | 0.35 |  |  | 0.86(0.40) | 0.0-4.7 | 0.78 | 0.53 |
| SO_2_ (ppb) | 5.66(2.88) | 0.0-26.71 | 5.17 | 3.47 |  | 3.99(2.01) | 1-29.5 | 3.58 | 2.46 |  |  | 4.94(2.51) | 0.13-27.24 | 4.46 | 2.93 |  |  | 7.83(3.42) | 0.0-33.87 | 7.29 | 4.25 |
| O_3_ (ppm) | 0.03(0.01) | 0.0-0.08 | 0.03 | 0.01 |  | 0.02(0.15) | 0.0-18.88 | 0.02 | 0.02 |  |  | 0.01(0.01) | 0.0-0.06 | 0.01 | 0.01 |  |  | 0.01(0.01) | 0-0.05 | 0.01 | 0.01 |

SD: Standard deviation

IQR: Interquartile range

**Table S3. Summary Statistics of Southern region for Temperature and other Meteorological Variables with the Level of Air pollutants by Season**

| **Parameter** | **Spring** | | | |  | **Summer** | | | |  |  | **Fall** | | | |  |  | **Winter** | | | |
| --- | --- | --- | --- | --- | --- | --- | --- | --- | --- | --- | --- | --- | --- | --- | --- | --- | --- | --- | --- | --- | --- |
|  | **Mean (SD)** | **Range** | **Median** | **IQR** |  | **Mean (SD)** | **Range** | **Median** | **IQR** |  |  | **Mean (SD)** | **Range** | **Median** | **IQR** |  |  | **Mean (SD)** | **Range** | **Median** | **IQR** |
| **Temperature** |  |  |  |  |  |  |  |  |  |  |  |  |  |  |  |  |  |  |  |  |  |
| Mean (°C) | 13.07(5.38) | -2.5-25.9 | 13.3 | 8.15 |  | 24.63(3.04) | 14.4-31.5 | 24.7 | 4.7 |  |  | 16.47(6.08) | -1.7-29.6 | 17.4 | 9.2 |  |  | 2.91(4.05) | -8-17.9 | 2.7 | 5.7 |
| Minimum (°C) | 8.2(5.5) | -8.3-20.1 | 8.7 | 8.3 |  | 21.33(3.15) | 8-28.2 | 21.5 | 4.7 |  |  | 12.34(6.75) | -6.9-26.8 | 13.1 | 10.4 |  |  | -1.21(4.39) | -15.3-15.5 | -1.4 | 6 |
| Maximum (°C) | 18.66(6.06) | 0.0-34.4 | 18.8 | 8.8 |  | 28.9(3.74) | 15.8-37.7 | 29.2 | 5.5 |  |  | 21.63(5.96) | 0.8-34.7 | 22.7 | 8.6 |  |  | 7.81(4.37) | -4.3-22.4 | 7.7 | 6.1 |
| Diurnal temperature (°C) | 10.46(4.06) | 1.1-25.7 | 10 | 5.8 |  | 7.57(3.0) | 0.8-20.4 | 7.3 | 4.1 |  |  | 9.29(3.44) | 1-22.4 | 9 | 4.8 |  |  | 9.02(3.31) | 1-23.1 | 8.7 | 4.1 |
| **Other meteorological variables** |  |  |  |  |  |  |  |  |  |  |  |  |  |  |  |  |  |  |  |  |  |
| Precipitation (mm) | 2.91(9.6) | 0.0-139.5 | 0.0 | 0.4 |  | 7.76(21.58) | 0.0-310 | 0.05 | 3.5 |  |  | 2.18(9.79) | 0.0-265 | 0.0 | 0.05 |  |  | 1.08(4.3) | 0.0-86 | 0.0 | 0.05 |
| Precipitation (log mm) | 0.18(1.3) | -3-4.94 | 0.0 | 0.0 |  | 0.46(1.74) | -3.0-5.74 | 0.0 | 1.25 |  |  | 0.02(1.2) | -3-5.58 | 0.0 | 0.0 |  |  | -0.11(1.14) | -3-4.45 | 0.0 | 0.0 |
| Relative humidity (%) | 58.79(16.35) | 17-99 | 58.8 | 24 |  | 75.09(11.19) | 32.1-99 | 75.95 | 14.6 |  |  | 65.36(13.87) | 20-99 | 66.1 | 18 |  |  | 55.32(17.59) | 16.1-99 | 54.8 | 27 |
| Sea-level pressure (hPa) | 1015(6.14) | 993.8-1031.2 | 1015.1 | 8.4 |  | 1008.01(4.17) | 996.9-1020.2 | 1008.1 | 5.9 |  |  | 1017.87(5.36) | 995.3-1033.8 | 1017.9 | 7 |  |  | 1022.86(5.13) | 998.5-1038.4 | 1023.1 | 6.7 |
| **Level of air pollutants** |  |  |  |  |  |  |  |  |  |  |  |  |  |  |  |  |  |  |  |  |  |
| PM10 (µg/m^3^) | 63.52(49.19) | 0.003-974 | 55 | 35.58 |  | 44.35(23.33) | 5.38-203.75 | 39.92 | 29.88 |  |  | 46.79(26.08) | 0.0-375.42 | 40.68 | 29.54 |  |  | 54.48(27.88) | 0.0-278.83 | 48.75 | 32.92 |
| NO_2_ (ppm) | 0.02(0.01) | 0.0-0.7 | 0.02 | 0.01 |  | 0.02(0.01) | 0.001-0.07 | 0.02 | 0.01 |  |  | 0.02(0.01) | 0.001-0.09 | 0.02 | 0.01 |  |  | 0.03(0.01) | 0.001-0.12 | 0.02 | 0.02 |
| CO (ppm) | 0.49(0.24) | 0.0-3.11 | 0.45 | 0.3 |  | 0.42(0.2) | 0.05-1.9 | 0.39 | 0.28 |  |  | 0.5(0.27) | 0.08-2.18 | 0.45 | 0.36 |  |  | 0.67(0.39) | 0.0-3.37 | 0.58 | 0.51 |
| SO_2_ (ppb) | 5.78(3.64) | 0.0-43.5 | 4.79 | 3.88 |  | 5.06(4.03) | 0.0-31.5 | 3.75 | 3.67 |  |  | 5.36(3.53) | 0.13-35.17 | 4.29 | 3.61 |  |  | 7.06(4.13) | 0.0-45.78 | 5.96 | 4.69 |
| O_3_ (ppm) | 0.03(0.01) | 0.003-0.09 | 0.03 | 0.01 |  | 0.02(0.01) | 0.001-0.07 | 0.02 | 0.02 |  |  | 0.02(0.01) | 0.001-0.08 | 0.02 | 0.01 |  |  | 0.02(0.01) | 0.0-0.04 | 0.01 | 0.01 |

SD: Standard deviation

IQR: Interquartile range
